# Impact of Mycotoxin Contaminations on Aquatic Organisms: Toxic Effect of Aflatoxin B1 and Fumonisin B1 Mixture

**DOI:** 10.3390/toxins14080518

**Published:** 2022-07-29

**Authors:** Davide Di Paola, Carmelo Iaria, Fabiano Capparucci, Alessia Arangia, Rosalia Crupi, Salvatore Cuzzocrea, Nunziacarla Spanò, Enrico Gugliandolo, Alessio Filippo Peritore

**Affiliations:** 1 Department of Chemical, Biological, Pharmaceutical, and Environmental Science, University of Messina, 98166 Messina, Italy; davide.dipaola@unime.it (D.D.P.); carmelo.iaria@unime.it (C.I.); fabiano.capparucci@unime.it (F.C.); alessiaarangia@gmail.com (A.A.); aperitore@unime.it (A.F.P.); 2 Department of Veterinary Science, University of Messina, 98166 Messina, Italy; rcrupi@unime.it (R.C.); egugliandolo@unime.it (E.G.); 3 Department of Pharmacological and Physiological Science, School of Medicine, Saint Louis University, Saint Louis, MO 63104, USA; 4 Department of Biomedical and Dental Sciences and Morphofunctional Imaging, University of Messina, 98166 Messina, Italy

**Keywords:** mycotoxins, combined toxicity, oxidative stress

## Abstract

(1) Background: Multiple contaminations of several mycotoxins have been detected in human and veterinary food and feed worldwide. To date, a number of studies on the combined effects of mycotoxins have been conducted on cell and animal models, but very limited studies have been done on aquatic organisms. (2) The purpose of the present study was to evaluate the combined toxic effects of Aflatoxin B1 (AFB1) and Fumonisin B1 (FB1) on zebrafish (*Danio rerio*) embryos. (3) Results: Our results showed that the combination of AFB1 and FB1 at nontoxic concentrations exerted a negative effect on the lethal endpoints analyzed, such as survival, hatching, and heart rate. In addition, the mixture of mycotoxins caused an increase in the levels of enzymes and proteins involved in the antioxidant process, such as superoxide dismutase (SOD) and catalase (CAT), both in terms of protein levels and gene expression, as well as an increase in the levels of the detoxification enzymes glutathione s-transferases (GST) and cytochromes P450 (CYP450). Furthermore, we showed that the mycotoxin mixture induced an increase in pro-apoptotic proteins such as bax and caspase 3, and at the same time reduced the gene expression of the anti-apoptotic bcl-2 protein. Finally, a significant decrease in thyroid function was observed in terms of triiodothyronine (T3), thyroxine (T4), and vitellogenin (VTG) levels. (4) Conclusion: We can say that the mixture of mycotoxins carries a greater risk factor than individual presences. There is a greater need for effective detoxification methods to control and reduce the toxicity of multiple mycotoxins and reduce the toxicity of multiple mycotoxins in feed and throughout the food chain.

## 1. Introduction

The increasing use of plant-based ingredients augments the risk of introducing mycotoxins into feed during production and storage [1]. Mycotoxins pose a growing danger to aquaculture, with the greatest risk to farmed fish [2]. As the metabolic byproducts of molds, mycotoxins can contaminate different types of foods and their processed products because they remain stable and survive food processing [3]. Aflatoxin B1 (AFB1), through feed contamination, not only poses a hazard to farmed fish but can also be dangerous to human health, given the ability of the pollutants to transmit through the food chain [4]. The aflatoxins, secondary metabolites of molds *Aspergillus flavus*, *Aspergillus parasiticus*, and, to a lesser degree, *Aspergillus nomius*, are a class of compounds linked to pyranocoumarin [5]. Several investigations have revealed that *Aspergillus* and AFB1 contamination in fish feed is widespread worldwide. Due to the hot and humid conditions that favor the development of microorganisms, tropical countries are among those with the highest risk of mycotoxin contamination [2]. For example, in Asia and Africa, the average levels of AFB1 in fish feed found were high, ranging from 51.83 μg/kg to a maximum of 220.61 μg/kg, compared with European countries that were negligible (0.43 μg/kg) [4,6,7,8,9]. In addition to aflatoxins, another important mycotoxin often found in food for human or animal consumption is fumonisin, a mycotoxin produced by *Fusarium* species [10,11]. Fumonisins are a family of structurally-related polar metabolites released by *Fusarium* fungi, in which fumonisin B1 (FB1) is the most toxic form and can mainly contaminate maize and maize products worldwide [12]. FB1 is associated with toxicity in several organs such as the liver and kidneys, as well as with immunotoxicity. It is classified by the International Agency for Research on Cancer (IARC, 2002) as a possible human carcinogen (2B) [13], along with AFB1, which was classified as carcinogenic to humans belonging to Group 1 of IARC. Mycotoxins can also be produced simultaneously by several toxin-producing fungi, and foods and feeds can be polluted by several fungal species simultaneously [14,15]. As a result, the natural co-occurrence of two or more mycotoxins is often detected in foods [16]. AFB1 was found together with FB1 in an area with a high incidence of primary human hepatocellular carcinoma, suggesting that the mixture may be involved in a synergistic mechanism of toxicity [17,18]. For this reason, this combination has received the most attention in the past decade. Several studies have shown how both AFB1 and FB1 cause increased expression of molecules involved in the oxidative stress pathway, like superoxide dismutase (SOD), catalases (CAT), and glutathione s-transferase (GST) that lead to the disruption of the cellular redox balance and thus result in oxidative stress-induced multiple tissue injury [19,20,21,22].

Animal models and cell-based analytical methods have been widely used to explore the combined effects of mycotoxins on humans [23]. Nonetheless, due to the existing limitations including immortalization, cell survival, or metabolic imbalance for cell-based assay—costly and time-consuming for traditional mammalian models—viable alternatives are being sought to study the effects of mycotoxins. Zebrafish (*Danio rerio*) are often used as a favorable organism for toxicology investigations because of their high fecundity. Therefore, it is an outstanding vertebrate model due to its highly developmental similarity to mammals [24,25]. The objective of this study was to evaluate the co-toxicity of AFB1 and FB1 on the zebrafish model, evaluating lethal and sub-lethal endpoints of mycotoxin mixture toxicity to induce oxidative stress and apoptosis.

## 2. Results

### 2.1. Survival Rate, Hatching Rate, and Morphology of AFB1 and FB1 Individual Exposures

To determine the most appropriate concentrations and time points for the following experiments, mycotoxin mixtures AFB1 at 0.01, 0.05, and 0.1 mg/kg, and FB1 at 0.1, 0.5, and 1 mg/kg were applied to observe the morphology, survival, and hatching rate of embryos/larvae (Table 1). Exposure to AFB1 showed no toxic effects on survival and morphological changes for concentrations of 0.01 and 0.5 mg/kg in embryos and larvae from 24 up to 96 h post-fertilization (hpf). A significant decrease compared with the control (CTRL) group was observed at a concentration of 0.1 mg/kg at 96 hpf. FB1 concentrations showed toxic effects at concentrations of 0.5 and 1 mg/kg with a high decrease in survival rate at 96 hpf. Toxic effects from AFB1 and FB1 exposure were also observed by analysis of the larval hatching rate. Hatching began between 48–72 hpf for the CTRL group, as well as for AFB1 concentrations 0.01 and 0.05 mg/kg. A significant delay in hatching was observed for AFB1 concentration of 0.1 mg/kg at 48, 72, and 96 hpf. FB1 exposure showed no toxic effect on hatching compared with the CTRL group at 0.1 mg/kg. FB1 exposure at 0.5 and 1 resulted in a delay from 48 hpf until 96 hpf compared with CTRL. Morphological deficits were seen only for the 0.1 mg/kg for AFB1 exposure at time points up to 96 hpf, such as in the spinal axis curve and pericardium (Table 1). FB1 exposure altered the normal development of embryos/larvae at 0.5 and 1 mg/kg, with scoliosis, pericardial, and yolk sac edema, but not for the 0.1 mg/kg.

### 2.2. Toxic Effect of Combined Exposure of AFB1 and FB1 on Malformation and Body Length

**Figure 1:**
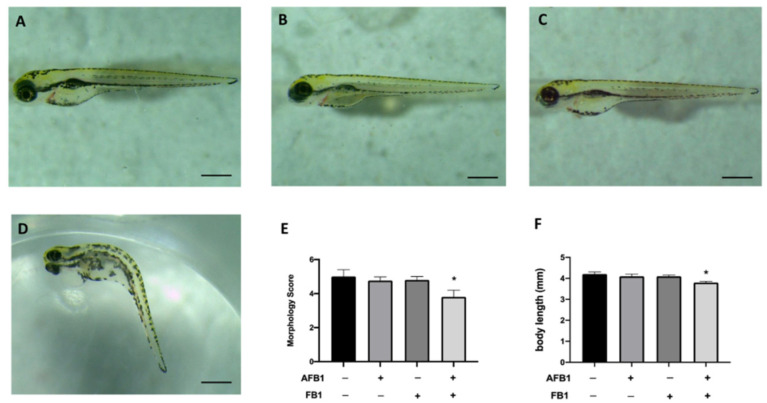
Morphology. Effects of AFB1 0.05 mg/kg, FB1 0.1 mg/kg and mixture on morphological changes (**A**), score and body length at 96 hpf. Larva at 96 hph of CTRL (**A**), AFB1 (**B**), FB1 (**C**), AFB1 + FB1 (**D**) groups. Morphology Score (**E**), body length (**F**) of zebrafish larvae treated. Data are expressed as means ± SEM; * *p* < 0.05 versus CTRL.

The toxic effects of mycotoxin mixtures were observed in malformations at 96 hpf. The morphology score of the single exposure AFB1 0.05 mg/kg and FB1 0.1 mg/kg group showed no significant change compared with CTRL. Moreover, when embryos were co-exposed with mycotoxin mixtures at the same concentrations as a single exposure, we observed developmental defects (Figure 1). Abnormalities, such as pericardial edema and scoliosis were found in the coexposure group at 96 hpf (Figure 1). In addition, we assessed larvae body length at 96 hpf to evaluate the degree of development (Figure 1). Exposure to the individual mycotoxins AFB1 and FB1 had no effect on body length at 96 hpf, whereas the mixture induced a short decrease, suggesting that modest concentrations of mycotoxins caused alterations in the normal structure of the larvae during development.

### 2.3. Effect of AFB1, FB1, and Mixture on Thyroid Function

**Figure 2:**
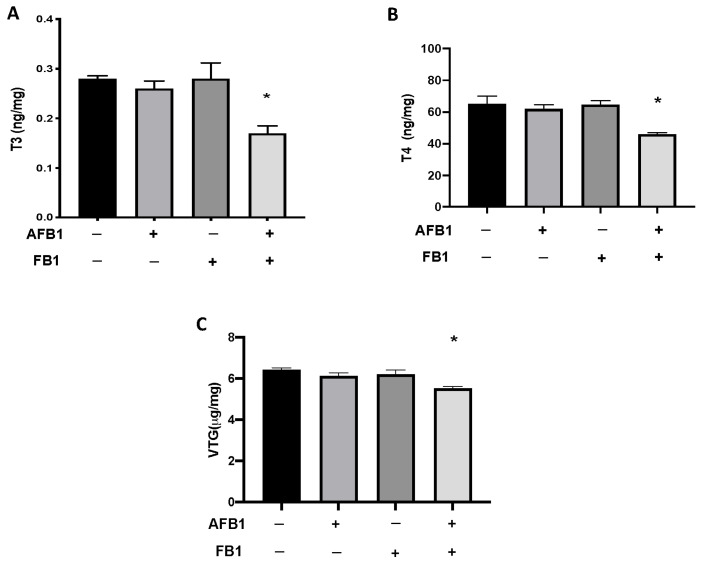
ELISA assay. Effects of exposure to AFB1 0.05 mg/kg, FB1 0.1 mg/kg singly and in mixture on thyroid hormones levels (**A**) Triiodothyronine (T3); (**B**) Thyroxine(T4), as well as (**C**) Vitellogenin (VTG), the egg yolk precursor protein important in early life stage development of embryo/larvae, levels at 96 hpf. Data are expressed as means ± SEM; * *p* < 0.05 versus CTRL.

We evaluated the levels of thyroid hormones (THs) that are very important in the early embryonic development of fish. Thyroid function was assessed through analysis of the levels of triiodothyronine (T3) and thyroxine (T4). Exposure to AFB1 0.05 mg/kg and FB1 0.1 mg/kg did not alter the levels of T3 and T4 compared with the CTRL group. Contrarily, the mycotoxins mixture (AFB1 0.05 mg/kg + FB1 0.1 mg/kg) significantly decreased both the T3 level and T4 concentration in the larvae at 96 hpf (Figure 2A,B). Moreover, we assessed the level of vitellogenin (VTG) at 96 hpf. We observed a decrease in the AFB1 and FB1 mycotoxin mixture groups compared with the CTRL groups. In contrast, its level was unchanged in the single exposure of AFB1 and FB1 compared with the control group (Figure 2C).

### 2.4. Toxic Effect of AFB1 and FB1 Mixture on Lipid Peroxidation and Stress Oxidative Pathway

**Figure 3:**
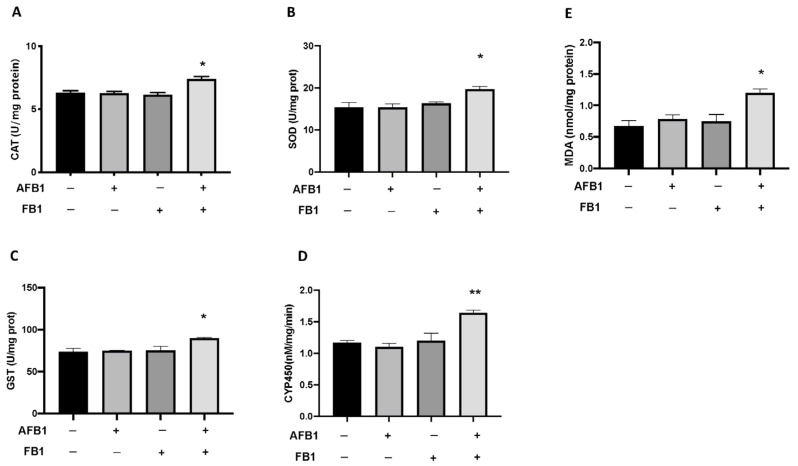
ELISA assay. Effects of exposure to AFB1 0.05 mg/kg, FB1 0.1 mg/kg singly and in mixture on antioxidant and detoxification enzymes activity at 96 hpf. CAT (**A**), SOD (**B**), GST (**C**), CYP450 (**D**). Content of malondialdehyde, a marker of lipid peroxidation, at 96 hpf after single and mixed exposure of AFB1 and FB1 (**E**). Data are expressed as means ± SEM; * *p* < 0.05 versus CTRL; ** *p* < 0.01 versus CTRL.

Correspondingly, the activity of SOD, CAT, as well as detoxification enzymes glutathione s-transferases (GST) and cytochromes P450 (CYP450) increased significantly in the larvae after the exposure to the mixture of mycotoxins AFB1 0.05 mg/kg and FB1 0.1 mg/kg compared with the control (Figure 3). The results in Figure 3 illustrate that the malondialdehyde (MDA) content in the larval zebrafish increased significantly after the exposure to the mycotoxin mixture at 96 h compared with the control group. There was no effect on increased MDA content after a single exposure to AFB1 and FB1 (Figure 3).

The results are expressed as the mean of data of three independent experiments. * *p* < 0.05, ** *p* < 0.01, versus CTRL.

### 2.5. Toxic Effects of AFB1 and FB1 on Oxidative Stress and Apoptosis-Related Genes Expression

**Figure 4:**
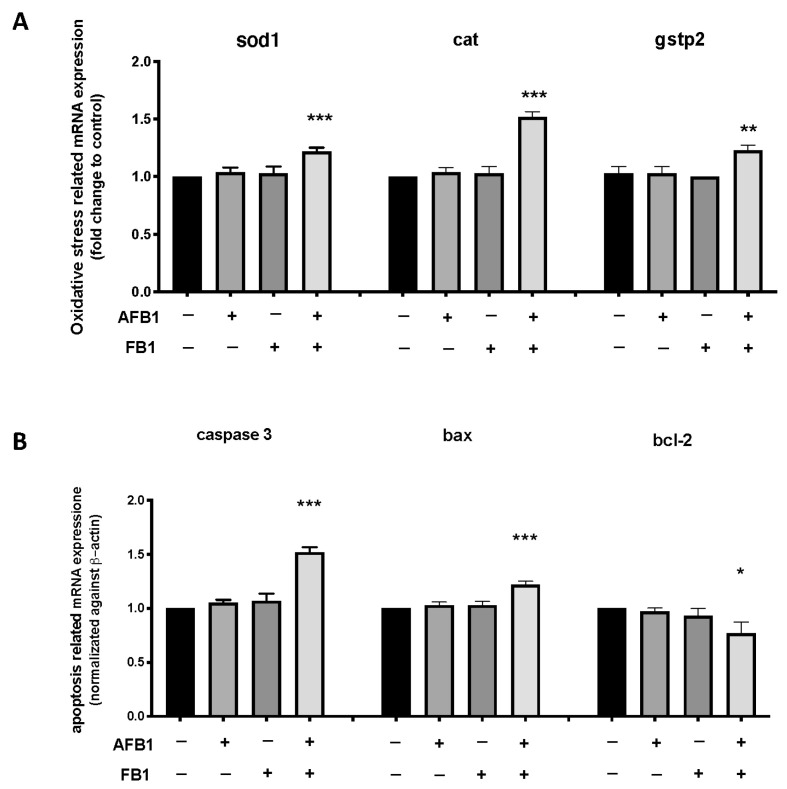
qPCR analysis. Effects of exposure to AFB1 0.05 mg/kg, FB1 0.1 mg/kg singly and in mixture on the expression of oxidative stress-related *sod1*, *cat*, and *gstp2* genes (**A**) and apoptosis-related caspase3, bax, and bcl-2 genes (**B**) at 96 hpf. The results are expressed as mean of data from three independent experiments. The expression levels of mRNA are represented as the fold change from the CTRL group. * *p* < 0.05, ** *p* < 0.01, *** *p* < 0.001 versus CTRL.

The RT-PCR results showed that the expression levels of the oxidative stress-related genes (*cat*, *sod1*, and *gstp2*) and apoptosis-related genes (casp3a, bcl2l1, and baxa) after exposure to AFB1, FB1, alone, and in combination. At the same time, the mycotoxin mixture significantly upregulated the expression levels of cat, sod, and gstp2 compared with the CTRL group. Contrarily, there were no significant changes in the expression levels of the antioxidant-gene for the AFB1 0.05 mg/kg and FB1 0.01 mg/kg alone exposure group (Figure 4). The mycotoxin mixture, at the same concentration of the single exposure groups, increased the mRNA expression levels of pro-apoptosis-related genes (caspase-3 and bax), at the same time causing a decrease in anti-apoptosis-related gene bcl-2 (Figure 4). On the contrary, the single exposure with AFB1 and FB1 did not increase the expression of apoptosis-related genes compared with the control group.

## 3. Discussion

One of the major issues for aquaculture productivity is the presence of mycotoxins in feed [26]. Because of the increasing frequency of simultaneous contamination with multiple mycotoxins, we focused on the potential co-toxicity between two in particular, AFB1 and FB1. To analyze a potential synergistic toxic action of these two mycotoxins, we first conducted a series of tests for exposure of AFB1 and FB1 individually at different concentrations, based on previous studies [27,28]. Furthermore, the concentrations of AFB1 tested in this study were very similar to those found in the feed, whereas for FB1 they were slightly lower than in the environment [25,29]. In addition, it has also been seen that these two mycotoxins may exhibit dose-dependent toxicity in zebrafish embryonic development, in which they have been shown to be teratogenic by increasing the incidence of malformations such as spinal lordosis, pericardial edema, narrowed head, and elongated heart, among others [30,31]. Our analysis of exposure to the individual mycotoxins, AFB1 and FB1, showed that there is a dose-dependent toxic response, early in embryonic development, in line with previous studies. In fact, we observed at 96 hpf alterations of the normal structure of the fish, such as curvature of the body axis and edema formation in the yolk sac or pericardium after dose-dependent exposure of single AFB1 and FB1.

Studies conducted on different experimental models, such as human cells in vitro or in vivo rat models, showed that exposure to AFB1 and FB1 alone or in combination can cause toxicity following prolonged exposure [30,32,33]. After investigating the different toxic and non-toxic concentrations of AFB1 and FB1, we assessed their potential co-toxic effects in early zebrafish development. Exposure to two concentrations, in themselves nontoxic, resulted in a slight, but significant, increase in malformations on larvae at 96 hpf, accompanied by a decrease in body length. In line with previous studies conducted on different models, we observed that exposure to a mixture of these mycotoxins, at a single nontoxic concentration, strongly affected the development of embryos, suggesting a potential hazard due to their co-presence even in cases of nontoxic concentrations.

Following early exposure to contaminants, a reduction in body length during zebrafish embryonic development was reported, accompanied by T4 and T3 level decreases [32,33]. THs make several contributions to scale formation and pigmentation during the transition from late larva to young zebrafish [34]. T4 and T3 are the major forms generated by the thyroid gland and play key roles in growth and development. Previous studies have shown that the presence of contaminants affecting T3 and T4 expression is not always equal after exposure to a mixture of mycotoxins [35,36]. Our data are in line with previous statements about a decrease in the ratio between T3 and T4 levels [37]. In our research, both T3 and T4 levels were markedly decreased after coexposure of AFB1 and FB1, but not after a single exposure. Thus, zebrafish tend to be affected by exogenous TH and thyroid inhibitors during the transition phase [34]. VTG is considered a biomarker of estrogen-associated endocrine disorders [38,39]. The VTG content was reduced in the mixture group, indicating that the two mycotoxins had an antiestrogenic effect. Conversely, the single mycotoxin group showed no difference regarding VTG, suggesting that the mixture of AFB1 and FB1 possessed the estrogenic effect.

Environmental stressors can immediately stimulate oxidative stress through a complex physiological process, and oxidative stress occurs when the level of reactive oxygen species is greater than the scavenging activities of antioxidants [40,41]. The toxic action of AFB1 is often associated with increased oxidative stress. Several studies have shown how AFB1 can cause increased oxidative stress when ingested in excessive amounts and repeatedly in fish models [31,42,43]. When toxic substances are metabolized by organisms, there are large amounts of reactive oxygen species (ROS), including superoxide, hydrogen peroxide, free radicals, hydrogen peroxide, and so on. The antioxidant enzyme system, such as SOD and CAT, mainly works to balance ROS and protect against oxidative damage in the organism by playing a key role in antagonizing oxidative stress [40,41]. SOD and CAT activities in the single mycotoxin exposure groups were not markedly changed, indicating that the determined concentration of a single mycotoxin was not sufficient to induce major oxidative stress. Given the wide presence of mycotoxins in foods, it is very necessary to clarify the mechanism of detoxification of mycotoxins. CYP450 and GST are two important factors involved in both human and animal detoxification mechanisms. Our present data show that CYP450 activity is increased in the following exposure to mycotoxin mixtures as opposed to single exposures of AFB1 and FB1. Therefore, CYP450 and GST had an important role in the toxic mechanism of AFB1 and FB1 on fish embryos. In addition, CYP450 and GST could play a crucial detoxifying role in the mixture of AFB1 and FB1 for *D. rerio*. Thus, the increases in SOD and CAT, as well as the detoxification enzymes GSH and CYP450 could be explained by the self-protection mechanism to antioxidative stress in fish [42]. In the present study, CAT and SOD activity were significantly higher after the mixture of AFB1 and FB1 than in the control group. By contrast, at 96 hpf a single exposure of AFB1 and FB1, respectively, did not show oxidative augmentation of CAT and SOD. Consequently, to confirm the involvement of the antioxidant defense in the mycotoxin’s mixture toxicity, we investigated the expressions of some antioxidant genes in this study. The expression of antioxidant genes is necessary to assess antioxidant capacity [35]. In our current study, exposure to AFB1 and FB1 alone did not increase the expression of *sod1*, *cat*, and *gstp2* at the mRNA level. The mixture of mycotoxins was able to increase the mRNA expression levels of oxidative stress-related genes, whereas the single exposure did not show this augmentation. Another important mechanism that contributes to impaired cell function under oxidative stress is lipid peroxidation. MDA is the primary by-product of lipid peroxidation (LPO), and an increase in its content embodies the degree of cellular damage caused by free radicals [43,44]. The content of MDA increased after exposure to the mixture, suggesting that the increase in LPO was inversely proportional to the antioxidant levels.

As a cell suicide mechanism, apoptosis can get rid of superfluous or undesired cells [45]. The caspase family has a critical function in cell apoptosis, and caspase-3 has been shown to be an important executor that is activated downstream in apoptosis pathways [46]. Previous studies have demonstrated the ability of AFB1 to activate the intrinsic apoptotic pathway, which is normally regulated by the activation of caspase-3 [47]. In our study, exposure to mixtures of AFB1 and FB1, but not to single exposures, leads to both the study of the induction of apoptosis accompanied by a significant increase in caspase-3 expression in line with what has been said previously [48]. The increase in oxidative stress caused the activation of caspase-3 and bax while reducing the expression of bcl-2 [49]. Our data showed that the exposure of AFB1 and FB1 together at nontoxic concentrations increased the expression of the apoptosis-inducing target genes and reduced the expression of the anti-apoptotic factor.

## 4. Conclusions

In conclusion, the presence of multiple mycotoxins simultaneously can be a huge risk factor for animal and human health and therefore an important object of interest for food safety. Combinations of AFB1 0.05 and FB1 0.1 mg/kg seem to have additive effects on zebrafish embryos to a greater extent than single exposures. Prominent changes in the activities of antioxidant mechanisms, as well as detoxification mechanisms, were found in the group exposed to mycotoxin mixture AFB1 + FB1, compared with the single exposure groups. Further confirmation of the toxic effect of the mixture of AFB1 and FB1 is given by the increase in markers related to the apoptotic mechanism, which is closely related to an augmentation of oxidative stress. In addition, the mycotoxin mixture of AFB1 and FB1 also showed an effect on thyroid functions, decreasing thyroid hormone levels and VTG contents, biomarkers of estrogen-associated endocrine disorders. Further studies will be needed to clarify the ecotoxicological risks of long-term exposure to these mycotoxin mixtures, and the potential molecular mechanism of the cross-link between the endocrine and antioxidant systems.

## 5. Materials and Methods

### 5.1. Zebrafish Maintenance and Embryo Collection

Wild type (WT) mature zebrafish with an age of 6 months were used to produce embryos. The University of Messina Center of Experimental Fish Pathology (Centro di Ittiopatologia Sperimentale della Sicilia, CISS, Messina, Italy) supplied zebrafish maintenance and a fertilized egg collection. The fish were fed dry and live food twice a day at a rate of 3% of their body weight (BW). Mature females and males were mated at a 2:1 ratio for successful reproduction. The eggs were collected the next day in a chamber of 28 °C; bleached and non-fertilized eggs were discarded. For the experiments, only embryos that had reached the blastula stage were employed. The FET (fish embryo toxicity) test was carried out in accordance with OECD guidelines [50] and ISO 15088.

### 5.2. Survival Rate, Hatching Rate, and Morphology Score

Healthy embryos were implanted in 24-well culture plates at 4 h post-fertilization (hpf) (1 embryo in 2 mL solution/well). Zebrafish embryos were exposed to AFB1 and FB1 for 24–96 hpf to measure the toxic effects over a continuing observation period. Fertilized eggs were transferred into 24-well plates with test solutions and incubated at 28 °C at a 14:10 h day/night light regime. Embryo medium was composed with 15 mM NaCl, 0.5 mM KCl, 1 mM CaCl_2_, 1 mM MgSO_4_, 0.15 mM KH_2_PO_4_, 0.05 mM Na_2_HPO_4_, and 0.7 mM NaHCO_3_ at pH 7.3. Briefly, embryos were exposed to water only (blank control); AFB1 at 0.01, 0.05, and 0.1 mg/kg, and FB1 at 0.1, 0.5, and 1 mg/kg were added to embryo water (60 replicates were used for each experimental group for each experiment, and the experiments were repeated three times). AFB1 (CAS:1162-65-8) and FB1 (CAS Number: 116355-83-0) were purchased from the Sigma-Aldrich Company (St. Louis, MO, USA) and Sangon Biotech (Shanghai, China) Co., Ltd. (China) respectively. The mycotoxins were dissolved in DMSO (AFB1) and Milli-Q water (FB1) for the stock solution and then reconstituted in the embryo medium. The solutions were changed daily and the entire survival rate and developmental abnormalities of embryos and larvae were monitored and photo-recorded at 24, 48, 72, and 96 hpf [51]. During the period of exposure to the substances, the embryos/larvae were observed every 24 h for abnormalities in development, survival, hatching, as well as morphological changes [52]. Morphology scores were determined at 96 hpf as previously described. Nine endpoints, including body shape, somites, notochord, tail, fins, heart, face, brain, and pharyngeal arches/jaws, were examined to evaluate the phenotypes of the zebrafish, and eight larval specimens per group were used for scoring [53].

### 5.3. Gene Expression Analysis

The total RNA from zebrafish larvae (20 per experimental group of each experiment) was homogenized and isolated in 0.50 mL TRIzol reagent (Invitrogen, Waltham, MA, USA) according to the manufacturer’s instructions. The ratio of absorbance at 260–280 nm, as well as RNA quality, was evaluated by gel electrophoresis, with the concentration measured with NanoDrop 2000 (Thermo Scientific, Waltham, MA, USA). An iScript RT-PCR kit (Bio-Rad, Hercules, California, Stati Uniti, #1708891) was used to synthesize first-strand cDNA according to the manufacturer’s recommendations. The reverse transcription master mix was prepared by adding 1 μg of RNA template to the iScript reverse transcription Supermix. Real-time PCR was performed following the manufacturer’s instructions (SsoFast EvaGreen Supermix, #1725202, Bio-Rad), adding 1 μL of cDNA to the mix for a final volume of 20 μL sample cDNA. PCR conditions were: initial denaturation at 95 °C for 15 min, followed by 45 cycles of amplification at 95 °C for 20 s and 60 °C for 40 s. Final extension at 60 °C for 60 s and a hold at 4 °C were then performed using a StepOnePlus Real-Time PCR System (Applied Biosystems, Waltham, MA, USA). All the qPCR reactions were performed with three parallel samples; the negative control contains no sample template. Primers detail information was reported in a previous study (sod1 ID: 3055; cat ID: 30068; gstp 2 ID 553169; bcl2l1 ID 114401; baxa ID: 58081; casp3a ID 140621) [54,55,56]. Data analysis was performed using the 2^−∆∆Ct^ method and the results are expressed as fold changes to the control group.

### 5.4. Determination of Oxidative Stress Markers and MDA

The larvae (20 per experimental group of each experiment, AFB1, FB1, and mixture) were homogenized (1:20, *w*/*v*) in 50 mmol L^−1^ potassium phosphate buffer (pH 7.0) containing 0.5 mmol L^−1^ EDTA, followed by centrifugation at 13,680× *g* for 30 min at 4 °C. The supernatant was collected and used for the assay of biochemical parameters. The indexes of oxidative stress like antioxidant enzymes including SOD (Nanjing Jiancheng Bioengineering Institute, NJBI, Cat A001-1-2), CAT (NJBI, Cat A007-2-1), and detoxification enzymes including CYP450 (NJBI, Cat 48T/96T) and GST (NJBI, Cat A004-1-1) were determined according to the instructions of the respective assay kits.

### 5.5. Thyroid Hormone Measurement

Levels of vitellogenin (VTG), triiodothyronine (T3), and thyroxine (T4) were determined using ELISA kits. Briefly, the larvae (20 per experimental group of each experiment, AFB1, FB1, and mixture) were sacrificed and homogenized in a 0.4 mL ELISA buffer by using an Ultra-Turrax T8 basic homogenizer (IKA, Staufen, Germany). Then, the structures in the samples were disrupted by intermittent sonic oscillation for 5 min on ice, and the samples were vortexed vigorously for 10 min and centrifuged at 5000× *g* for 10 min at 4 °C. The supernatant was collected and stored at −80 °C until analysis. VTG (Creative Diagnostic, New York, NY, USA; Product No. DEIA5068), T3 (Uscnlife, Wuhan, China; Product No. CEA453Ge), and T4 (ASPIRA CHEMICAL, Oakland, CA, USA; Product No. 815961) were measured by an enzyme-linked immunosorbent assay following the manufacturer’s recommendation (Uscnlife, Wuhan, China).

### 5.6. Statistical Evaluation

For multiple comparisons, a two-way/one-way ANOVA was used, followed by a Bonferroni post-hoc test. GraphPad Prism 8 was used for statistical analysis. Statistically significant differences in the mean are indicated by asterisks.

## Figures and Tables

**Table 1:** Individual exposure of AFB1 and FB1 effects on zebrafish larvae endpoints.

	Survival				Hatching			Morphology
	24 h	48 h	72 h	96 h	48 h	72 h	96 h	96 h
CTRL	98.33 ± 0.88	98.00 ± 1	97.67 ± 1.20	97.67 ± 1.20	22.67 ± 1.45	100 ± 0	100 ± 0	-
AFB1 0.01 mg/kg	98.33 ± 0.88	97.33 ± 0.88	97.00 ± 1	97.00 ± 1	22.33 ± 1.45	99.67 ± 0.33	99.67 ± 0.33	-
AFB1 0.05 mg/kg	98.33 ± 0.88	97.67 ± 0.66	97.33 ± 0.88	97.33 ± 0.88	22 ± 1.73	99 ± 0.33	99.3 ± 0.57	-
AFB1 0.1 mg/kg	99.00 ± 0.57	95.00 ± 0.57 **	86.33 ± 0.88 ***	83.00 ± 1.15 ***	18.67 ± 1.76 **	90.00 ± 0.57 ***	92.00 ± 0.57 ***	SC, PE
FB1 0.1 mg/kg	98.33 ± 0.88	98.00 ± 1	97.67 ± 1.20	97.67 ± 1.20	22.33 ± 1.45	99.67 ± 0.33	99.67± 0.33	-
FB1 0.5 mg/kg	98.33 ± 0.88	96.00 ± 0.57	93.00 ± 01.52 ***	90.33 ± 1.20 ***	3 ± 1.15 ***	86.00 ± 1.15 ***	94 ± 1.15 ***	SC
FB1 1 mg/kg	99.00 ± 0.57	95.00 ± 0.57 **	88.33 ± 1.45 ***	83.33 ± 0.88 ***	2.33 ± 0.33 ***	80.67 ± 1.52 ***	93.00 ± 1.73 ***	SC, PE, YE

Lethal endpoint at 96 hpf after exposure of different concentration of AFB1 (0.01, 0.05, and 0.1 mg/kg) and FB1 (0.1, 0.5, and 1 mg/kg). Survival rate, hatching rate, and morphological alteration (SC = Scoliosis, PE = Pericardial Edema, YE = Yolk Sac Edema) were observed and reported as means ± SEM (standard error of mean); ** *p* < 0.01; *** *p* < 0.001 versus CTRL.

## Data Availability

Not applicable.
