# Correction: Di Paola et al. Impact of Mycotoxin Contaminations on Aquatic Organisms: Toxic Effect of Aflatoxin B1 and Fumonisin B1 Mixture. *Toxins* 2022, *14*, 518

**DOI:** 10.3390/toxins17050246

**Published:** 2025-05-15

**Authors:** Davide Di Paola, Carmelo Iaria, Fabiano Capparucci, Alessia Arangia, Rosalia Crupi, Salvatore Cuzzocrea, Nunziacarla Spanò, Enrico Gugliandolo, Alessio Filippo Peritore

**Affiliations:** 1 Department of Chemical, Biological, Pharmaceutical, and Environmental Science, University of Messina, 98166 Messina, Italycarmelo.iaria@unime.it (C.I.); fabiano.capparucci@unime.it (F.C.); alessiaarangia@gmail.com (A.A.); aperitore@unime.it (A.F.P.); 2 Department of Veterinary Science, University of Messina, 98166 Messina, Italy; rcrupi@unime.it (R.C.); egugliandolo@unime.it (E.G.); 3 Department of Pharmacological and Physiological Science, School of Medicine, Saint Louis University, Saint Louis, MO 63104, USA; 4 Department of Biomedical and Dental Sciences and Morphofunctional Imaging, University of Messina, 98166 Messina, Italy

## Error in Figure

In the original publication [1], there was an image in Figure 1, FB1 group (C), that is similar to another present in another piece of work [2]. The authors have checked all the data in our lab and found the images on a database. The authors apologize for any inconvenience caused by this similarity. These errors do not affect the results or conclusions published in the article. The new, corrected Figure 1 is shown below. The authors state that the scientific conclusions are unaffected. This correction has been approved by the Academic Editor. The original publication has also been updated.

## Figures and Tables

**Figure 1:**
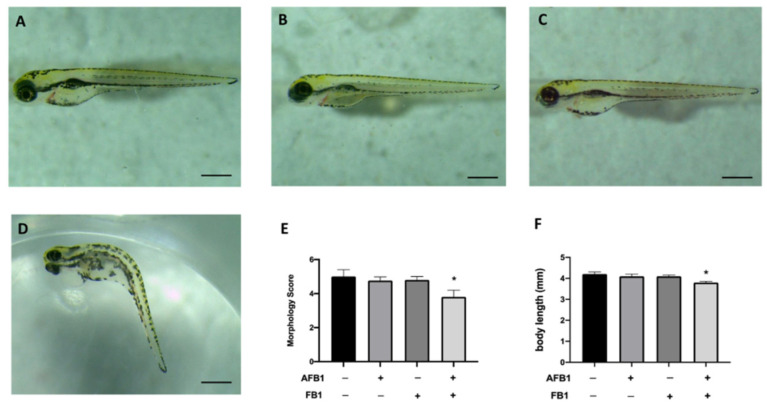
Morphology. Effects of AFB1 0.05 mg/kg, FB1 0.1 mg/kg and mixture on morphological changes (**A**), score and body length at 96 hpf. Larva at 96 hph of CTRL (**A**), AFB1 (**B**), FB1 (**C**), AFB1 + FB1 (**D**) groups. Morphology Score (**E**), body length (**F**) of zebrafish larvae treated. Data are expressed as means ± SEM; * *p* < 0.05 versus CTRL.
